# Supplementary material for: Mic19 depletion impairs endoplasmic reticulum-mitochondrial contacts and mitochondrial lipid metabolism and triggers liver disease
Source: Nat Commun. 2024 Jan 2;15:168. doi: 10.1038/s41467-023-44057-6 (PMC10762189; doi:10.1038/s41467-023-44057-6)
Supplement: Supplementary file 1 — Supplementary Information [file 41467_2023_44057_MOESM1_ESM.pdf]

SUPPLEMENTAL INFORMATION

Materials and Methods

Cell lentiviral transduction

To establish Mic19 overexpression and depleted cell lines, cells were infected with control, Mic19 specific overexpression or shMic19-encoded lentivirus. Mic19 was overexpressed or silenced in cells were performed as previously described[1].

Antibodies

The following primary antibodies were used in this study: Mic19 (Proteintech, 25625-1-AP), Mic60 (Proteintech, 10179-1-AP), Mic10 (Origene, TA505025), Sam50 (Abclonal, A3401), PDI (Santa Cruz, sc-20132), IP3R-III(BD, AB_397705), EMC2 (Proteintech, 25443-1-AP), SLC25A46 (Proteintech, 12277-1-AP), Flag (Sigma-Aldrich, F1804), GAPDH (Santa Cruz, sc-166545), Mic13 (Sigma-Aldrich, SAB1102836), Tom40 (Proteintech, 18409-1-AP), β-Tubulin (GNI, GNI4110-BT), LONP1 (Proteintech, 15440-1-AP), ClpP (Proteintech, 15698-1-AP), HSP60 (Abclonal, A0969), SOD2 (Proteintech, 24127-1-AP), Atf6 (Proteintech, 24169-1-AP), Chop (Cell Signaling Technology, #5554), GRP78 (Proteintech, 11587-1-AP), eIF2α (Cell Signaling Technology, #5324), phospho-eIF2α(Ser-51) (Cell Signaling Technology, #3398), ACC1 (Proteintech, 21923-1-AP), phospho-ACC1 (-S79) (Abclonal, AP0298); FASN (Proteintech, 10624-2-AP), CLS1 (Proteintech, 51055-1-AP), TAZ (Proteintech, 23306-1-AP), NDUFB6 (Proteintech, 16037-1-AP), SDHA (Proteintech, 14865-1-AP), UQCRC2 (Proteintech, 14742-1-AP), COX2 (Proteintech, 55070-1-AP), ATP5A1 (Proteintech, 14676-1-AP).

Histological analysis and immunochemistry staining

Mice were anesthetized with intraperitoneal injection of sodium pentobarbital (50 mg/kg) and sacrificed. The livers were dissected and cleaned with PBS (pH 7.4), followed by 4% paraformaldehyde in PBS overnight at 4°C. Then liver sections were stained with hematoxylin and eosin (H&E) or prepared for immunochemistry staining. For immunochemistry staining, after quenching the endogenous peroxidases with 3% hydrogen peroxide and blocking unspecific binding with 2% bovine serum albumin, sections were stained with the indicated antibody overnight at 4 °C. After extensive washing, sections were incubated with respective biotinylated secondary antibodies. Positive staining was visualized using DAB Substrate (Solarbio) following the ABC Kit (Agilent, MX-200-4).

Detection of cardiolipin level

Cardiolipin content was determined by CL-specific fluorescent dye 10-N-nonyl-acridine orange (NAO) staining. In brief, the objective cells were firstly fixed with 2% PFA at room temperature. After washing several times, cells were incubated with 1 μM NAO for 10 min. Finally, the fluorescence intensity was detected by flow cytometry analysis (Beckman Coulter). The data were analyzed by FlowJo 7.6.1 (Tree Star, San Carlos, USA).

RNA extraction, quantitative RT-PCR and RNA sequencing analysis

Mice were anesthetized with intraperitoneal injection of sodium pentobarbital (50 mg/kg) and sacrificed. The livers were immediately dissected and frozen for RNA isolation. Total RNA was extracted with TRI reagent (Sigma-Aldrich, T9424) and treated with DNase I (Thermo Fisher, 18068015). For cDNA synthesis, 2 μg of total RNA were reverse-transcribed using the RevertAid Synthesis Kit (Thermo Scientific, K1691). mRNA levels were quantified using SYBR Green Premix (ABclonal, RK21203). The relative changes of mRNA level were normalized to GAPDH, calculated using the 2-ΔΔCT method. All primers used in this study are listed in "Supplementary Table 1".

RNA-sequencing was performed to analyze the transcriptomics of Mic19$^{flox/flox}$ (control) and Mic19 LKO mice. Briefly, 5 μg of total RNA was prepared as initiation and then performed using Illumina TruSeq library construction kit, according to the manufacturer's protocols. Next, mRNA-seq libraries were sequenced using HiSeq2000 for 100-bp paired-end sequencing. Sequentially, raw mRNA-sequencing data were monitored using Fatsqc, followed by low quality bases were trimmed. Then data were mapped to hg19 genome reference by Tophat2 and a maximum of 2 mismatches were allowed. Differentially expressed genes (DEGs) were assessed using cufflinks. Gene ontology (GO) analysis was performed using KOBAS website.

Metabolic studies and blood bioassays

Relative metabolic studies of mice were performed as follows. Mice were anesthetized and their blood, liver and muscle samples were collected. Blood was drawn from the abdominal inferior vena cava, and was centrifuged (2000 × g, 15 min, 4 °C) to obtain serum. Serum levels of AST (aspartate aminotransferase, Nanjing Jiancheng, C010-2-1), ALT (alanine aminotransferase, Nanjing Jiancheng, C009-2-1), FFA (free fatty acids, Nanjing Jiancheng, A042-2-1), βHB (β-Hydroxybutyrate, Biovision, #K632-100), ALP (alkaline phosphatase, Nanjing Jiancheng, A059-2-2), γ-GT (gamma-glutamyl transpeptidase, Nanjing Jiancheng, C017-2-1), VLDL

(very low density lipoprotein, Nanjing Jiancheng, H249), were quantified according to the directions of the manufacturers, respectively. To quantify the concentrations of TG (triglyceride) of tissues, liver and muscle homogenate were obtained and determined by using an analytical kit (Nanjing Jiancheng, A110-1-1). Cholesterol (Nanjing Jiancheng, A111-1-1), acetyl-CoA (Sigma-Aldrich, MAK039-1KT), hydroxyproline (Nanjing Jiancheng, A030-2-1) contents in mouse livers were measured using the indicated assay kits according to the manufacturer's protocol.

Energy metabolism of mice

To investigate the whole-body energy metabolism of the mice, 9-week-old of Mic19$^{flox/flox}$ (control) and Mic19 LKO mice were individually housed in a comprehensive laboratory animal monitoring system (CLAMS) for 3 days. Before the experiment, mice body weights were measured. The carbon dioxide production (VCO2) and oxygen consumption (VO2) were detected with Oxymax apparatus system. VCO2 was calculated as divide the weight by the difference between output and input carbon dioxide flows. Similarly, VO2 was calculated as divide the weight by the difference between input and output oxygen flows. All data from the final day were analyzed.

Isolation of hepatic mitochondria

Liver mitochondrial fractions were isolated by sucrose density-gradient centrifugations according to the protocol[2]. In brief, mice were sacrificed with cervical dislocation and the livers were surgically removed. Next, approximate 0.75 g of liver was collected to Teflon potter (Sigma-Aldrich, D9063-1SET) in ice-cold buffer I (225 mM mannitol, 75 mM sucrose, 0.5% BSA, 0.5 mM EGTA and 30 mM Tris-HCl pH 7.4, 4 mL buffer per gram of liver) for homogenization. Mitochondria were isolated by gradient centrifugation of the liver homogenate. Firstly, the homogenate was centrifuged at 740× g for 5 min at 4 °C for twice to remove unbroken cellular debris and nucleus. Secondly, the supernatant was centrifuged at 9,000 × g for 10 min at 4 °C to obtain precipitated mitochondrial pellets. Thirdly, mitochondrial pellets were suspended in fresh ice-cold buffer II (225 mM mannitol, 75 mM sucrose, 0.5% BSA and 30 mM Tris-HCl pH 7.4), followed by centrifuged at 10,000× g for 10 min at 4 °C. Subsequently, fresh ice-cold buffer III (225 mM mannitol, 75 mM sucrose and 30 mM Tris-HCl pH 7.4) was added to mitochondrial pellets and the same centrifugation as above was conducted to obtain crude mitochondria fractions. Finally, the crude mitochondria fractions were resuspended with ice-cold MRB (250 mM mannitol, 5 mM HEPES (pH 7.4) and 0.5 mM EGTA) and purified by

ultra-centrifuged with percoll gradients.

BN-PAGE analysis

Solubilization of mitochondria for BN-PAGE were conducted as described previously[1]. Briefly, hepatic mitochondria were resuspended in ice-cold solubilization buffer A (50 mM sodium chloride, 50 mM Imidazole/HCL, 2 mM 6-aminohexanoic acid, and 1 mM EDTA, pH 7.0) and 4% digitonin was added for 13 min. Then, the lysates were centrifuged at 12,000 g for 10 min at 4 °C to collect the supernatant. Subsequently, proteins from mitochondrial homogenates were separated on 4~ 14% blue native polyacrylamide gels. Afterward, proteins were blotted to a PVDF membrane (Merck Millipore) following incubation with antibodies were as same as SDS-PAGE.

ROS measurement

Liver tissue sections from 3-month-old Mic19$^{flox/flox}$ (control) and Mic19 LKO mice were sectioned in slices (8μm), and incubated with MnTBAP 150μM at room temperature for 1h. Then the samples were stained with DHE (5μM) at 37°C for 30min, and counterstained with DAPI. Subsequently, the sections were analyzed by confocal microscope. The fluorescence intensity was analyzed by using ImageJ software.

Lipidomic analysis

Lipidomic analysis was performed as previously described[3, 4]. Briefly, 700 mg of livers were collected to obtain pure mitochondria. Next, the pre-cooled chloroform methanol mixed solution (2:1) was added. The mitochondria were Grinded and centrifuged at 12,000 × g at room temperature for 5 min. Lower layer fluid was transferred into a new tube and concentrated. Then the samples were dissolved in isopropanol and filtered for LC-MS. Lipid extracts were analyzed on an ACQUITY UPLC BEH C18 (100 × 2.1 mm, 1.7 μm, Waters) column at 50°C. The gradient elution of analytes was conducted with A. ultrapure water (60:40, 0.1% formic acid, 10 mM ammonium formate) and B. LC/MS grade acetonitrile/isopropanol (90:10, 0.1% formic acid, 10 mM ammonium formate) at 0.2 mL/min. After equilibration, 2 μL of each sample was injected. The lipid profiling was executed using ESI mode, and the data were collected at mass range of m/z 150~2,000. Data acquisition were performed with HCD scan. Finally, the data were processed and normalized. Lipid species identification was carried out using an internal spectral library.

Membrane potential measurement

For membrane potential detection, the HeLa cells were incubated with 200 nM Tetramethylrhodamine (TMRM) with DMEM at 37°C for 15 min. The indicated cells were washed three times with PBS and the fluorescence intensity of 10^4 cells was analyzed by flow cytometer (Beckman Coulter) with PE-A channel. The data were analyzed by FlowJo 7.6.1 (Tree Star, San Carlos, USA).

Mito-Keima mitophagy assay

The mito-Keima stabilized transient HeLa cell lines were constructed as reported previously [6]. Briefly, HeLa was infected twice with a lentiviral plasmid containing mito-Keima, and then cells were laid down in 96-well plates by the limited dilution method. Two weeks later, under a fluorescence microscope, the monoclones of mito-Keima stabilized transient HeLa cell lines were picked based on fluorescence. Next, the mito-Keima stabilized transient HeLa control and Mic19 ko cell lines were constructed. And the mito-Keima were excited with 458 nm and 561 nm laser and imaged by Leica SP8 confocal microscopy.

Measurement of hepatic oxygen consumption rates

The fresh liver tissues were homogenized in Miro5 Buffer (0.5mM EGTA, 3 mM MgCl2, 3 mM lactobionic acid, 20 mM taurine, 10 mM KH2PO4, 20 mM HEPES, 110 mM D-sucrose, 1 g/l essentially fatty acid free BSA, pH 7.1). 4 mg of homogenized liver tissues were load into the Oroboros O2k system. The hepatic oxygen consumption rates were measured before and after specific substrates and inhibitors were added in the following order: 1) 2.5 mM pyruvate, 10 mM glutamate, and 1 mM malate; 2) 2.5 mM ADP; 3) 125 μg/ml cytochrome c; 4) 5 mM succinate; 5) CCCP; 6) 200 nM rotenone; 7) 2 mM ascorbate, 0.5 mM TMPD.

DNA extraction and genotype identification

Isolation of genomic DNA of mice from mouse tails were conducted with alkaline lysis method. The lysis buffer is a mixture of solution consist of EDTA and NaOH. Then the tails were boiled for 1 hour on metal bath at 105°C. Subsequently, equal volume of 50mM Tris- HCl (pH 5.5) was added. Centrifugation at 12,000 × g for 2 min and the supernate was the template for genotype identification.

Adeno-associated virus production and transduction

Adeno-associated virus (AAV) production and purification were performed according to the protocol. Briefly, HEK293A cells were co-transfected the Mic19-Flag AAV shuttle plasmid together with Adenovirus helper plasmid, Rep/Cap packaging plasmid using PEI. 24 hours post transfection, prewarmed fresh medium was carefully added. 48 hour later, cells were harvested and freeze-thaw for five cycles. Then benzonase (Sigma- Aldrich, E1014) was added and incubate at 37°C for 30 min. After centrifugation at 4000 rpm for 30 minutes at 4°C, clarified supernatant was collected. Next, the supernatant was carefully added on top of the iodixanol gradient (15%, 20%, 40%, 60%). And the sample was centrifuged at 48,000 × g using a Beckman type 70Ti rotor for 1 hour at 18°C. Finally, the fraction in the 40% iodixanol was collected. Real-time PCR was performed to determine virus titer. To re-express Mic19 in the liver, each mouse (8-week-old) was infused with 1×10^{11} IFU virus particles through tail vein. After six weeks or 5 months of virus infection, the expression of Mic19 and other physiological indexes were measured.

REFERENCES

1. Tang, J. *et al.* Sam50-Mic19-Mic60 axis determines mitochondrial cristae architecture by mediating mitochondrial outer and inner membrane contact. *Cell Death Differ.* **27**, 146-160 (2020).
2. Wieckowski, M.R., Giorgi, C., Lebiedzinska, M., Duszynski, J. & Pinton, P. Isolation of mitochondria-associated membranes and mitochondria from animal tissues and cells. *Nat Protoc* **4**, 1582-1590 (2009).
3. Narváez-Rivas, M. & Zhang, Q. Comprehensive untargeted lipidomic analysis using core-shell C30 particle column and high field orbitrap mass spectrometer. *J. Chromatogr. A.* **1440**, 123-134 (2016).
4. Dasilva, G., Muñoz, S., Lois, S. & Medina, I. Non-Targeted LC-MS/MS Assay for Screening Over 100 Lipid Mediators from ARA, EPA, and DHA in Biological Samples Based on Mass Spectral Fragmentations. *Molecules* **4** (2019).
5. Hernández-Alvarez, M.I. *et al.* Deficient Endoplasmic Reticulum-Mitochondrial Phosphatidylserine Transfer Causes Liver Disease. *Cell* **177**, 881-895.e817 (2019).
6. Shu, L. *et al.* ATAD3B is a mitophagy receptor mediating clearance of oxidative stress-induced damaged mitochondrial DNA. *The EMBO Journal* **40** (2021).

Supplementary Table 1. Primers utilized for quantitative RT-PCR analysis.

Gene name	Symbol	Sequence
Carnitine Palmitoyltransferase 1A	*Cpt1a*	Forward: 5′-CTGAGCCATGAAGCCCTCAA-3′<br>Reverse: 5′-CACACCCACCACCACGATAA-3′
Carnitine Palmitoyltransferase 2	*Cpt2*	Forward: 5′- CCCAAACCCAGTCGTGATGA -3′<br>Reverse: 5′- TGTGCCTGGATTTCTGAGGG -3′
Acyl-CoA Dehydrogenase Family Member 9	*Acad9*	Forward: 5′- TCCAGAGGTCAGTCAACATGA -3′<br>Reverse: 5′- CCTGGTCAATTTTTCGAGAGTCC -3′
Acyl-CoA Dehydrogenase Short Chain	*Acads*	Forward: 5′- TTGCCGAGAAGGAGTTGGTC -3′<br>Reverse: 5′- AGGTAATCCAAGCCTGCACC -3′
Glyceraldehyde-3-Phosphate Dehydrogenase	*GAPDH*	Forward: 5′-GTTCCAGCACATTTTGCGAGT-3′<br>Reverse: 5′-GGTGAGGTCGATGTCTGCTT-3′
3-Hydroxy-3-Methylglutaryl-CoA Synthase 2	*Hmgcs2*	Forward: 5′- GAAGAGAGCGATGCAGGAAAC -3′<br>Reverse: 5′- GTCCACATATTGGGCTGGAAA -3′
3-Hydroxy-3-Methylglutaryl-CoA Lyase	*Hmgcl*	Forward: 5′- CAGGTGAAGATCGTGGAAGTC -3′<br>Reverse: 5′- GGAGCCCTGCTTCGGAAAC -3′
Collagen Type I Alpha 1 Chain	*Col1a1*	Forward: 5′- AGCACGTCTGGTTTGGAGAG -3′<br>Reverse: 5′- ACATTAGGCGCAGGAAGGTC -3′
Collagen Type III Alpha 1 Chain	*Col3a1*	Forward: 5′- AGCCACCTTGGTCAGTCCTA -3′<br>Reverse: 5′- GTGTAGAAGGCTGTGGGCAT -3′
CD68 Molecule	*Cd68*	Forward: 5′- TGTCTGATCTTGCTAGGACCG -3′<br>Reverse: 5′- GAGAGTAACGGCCTTTTTGTGA -3′
C-X-C Motif Chemokine Ligand 10	*Cxcl10*	Forward: 5′- CCAAGTGCTGCCGTCATTTTC -3′<br>Reverse: 5′- GGCTCGCAGGGATGATTTCAA -3′
Tumor Necrosis Factor	*Tnf*	Forward: 5′- TAGCCCACGTCGTAGCAAAC -3′<br>Reverse: 5′- GCAGCCTTGTCCCTTGAAGA -3′
CD36 Molecule	*Cd36*	Forward: 5′- ATGGGCTGTGATCGGAACTG -3′<br>Reverse: 5′- GTCTTCCCAATAAGCATGTCTCC -3′

SUPPLEMENTARY FIGURES

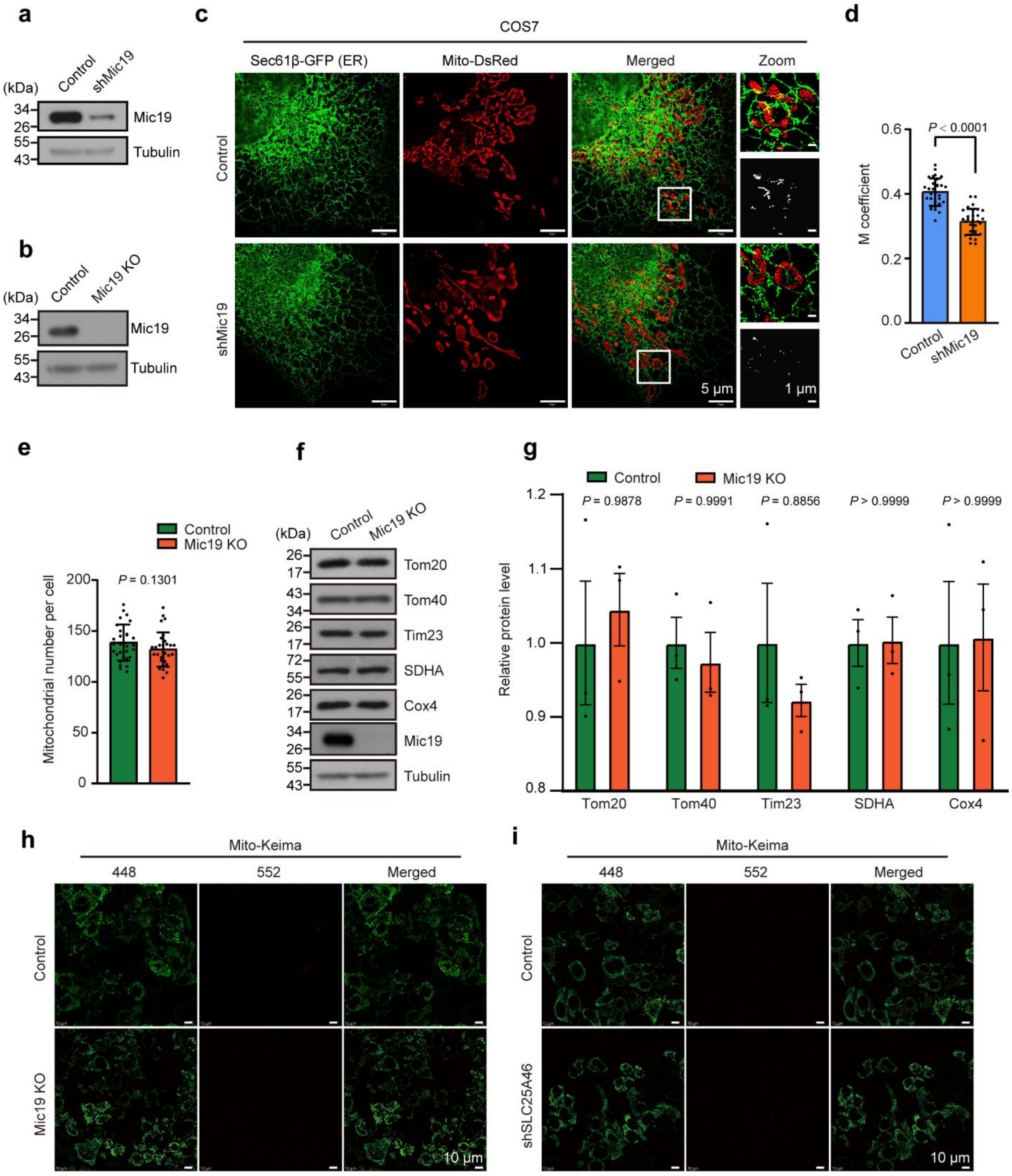

Figure S1. Mic19 depletion impairs ER-mitochondria contacts.

a HeLa cells were infected with control or Mic19 lentiviral particles. 5 days later, cell lysates were analyzed by Western blotting with anti-Mic19 or anti-Tubulin antibodies. **b** Control and Mic19 KO HeLa cell lysates were analyzed by Western blotting with antibodies against Mic19 or Tubulin. **c-d** COS7 cells stably expressing Mito-DsRed (a mitochondrial marker, red) and Sec61β-GFP (an ER marker, green) were infected with control (scrambled shRNA) or shMic19 lentiviral particles. 5 days later, cells were analyzed and imaged by HIS-SIM for co-localization

of mitochondria and ER (**c**). Quantitation of co-localization of ER and mitochondria in "**c**" was further analyzed by ImageJ software (**d**). n = 30 mitochondria were analyzed in 3 independent experiments. Data are presented as mean ± SD, statistical significance was assessed by two-tailed Student's t-test. **e** The mitochondria in control or Mic19 KO HeLa cells were imaged by confocal microscopy. Mitochondrial number per cell (n = 30 mitochondria) were further analyzed by ImageJ software. The data are presented as mean ± SEM, statistical significance was assessed by two-tailed Student's t-test. **f-g** Control and Mic19 KO HeLa cell lysates were analyzed by Western blotting with antibodies against Tom20, Tom40, Tim23, SDHA, Cox4, Mic19 or Tubulin (**f**). Representative immunoblots were from n = 3 independent experiments. Relative protein levels were further evaluated by densitometry analysis using ImageJ software (**g**). Data are presented as mean ± SD, statistical significance was assessed by two-way ANOVA. **h-i** Control, Mic19 KO or shSLC25A46 cells expressing Mito-Keima were detected for mitophagy. The mito-Keima were excited with 458 nm and 561 nm laser and imaged by confocal microscopy. *P* values are indicated in the figure. Source data are provided as a Source Data file.

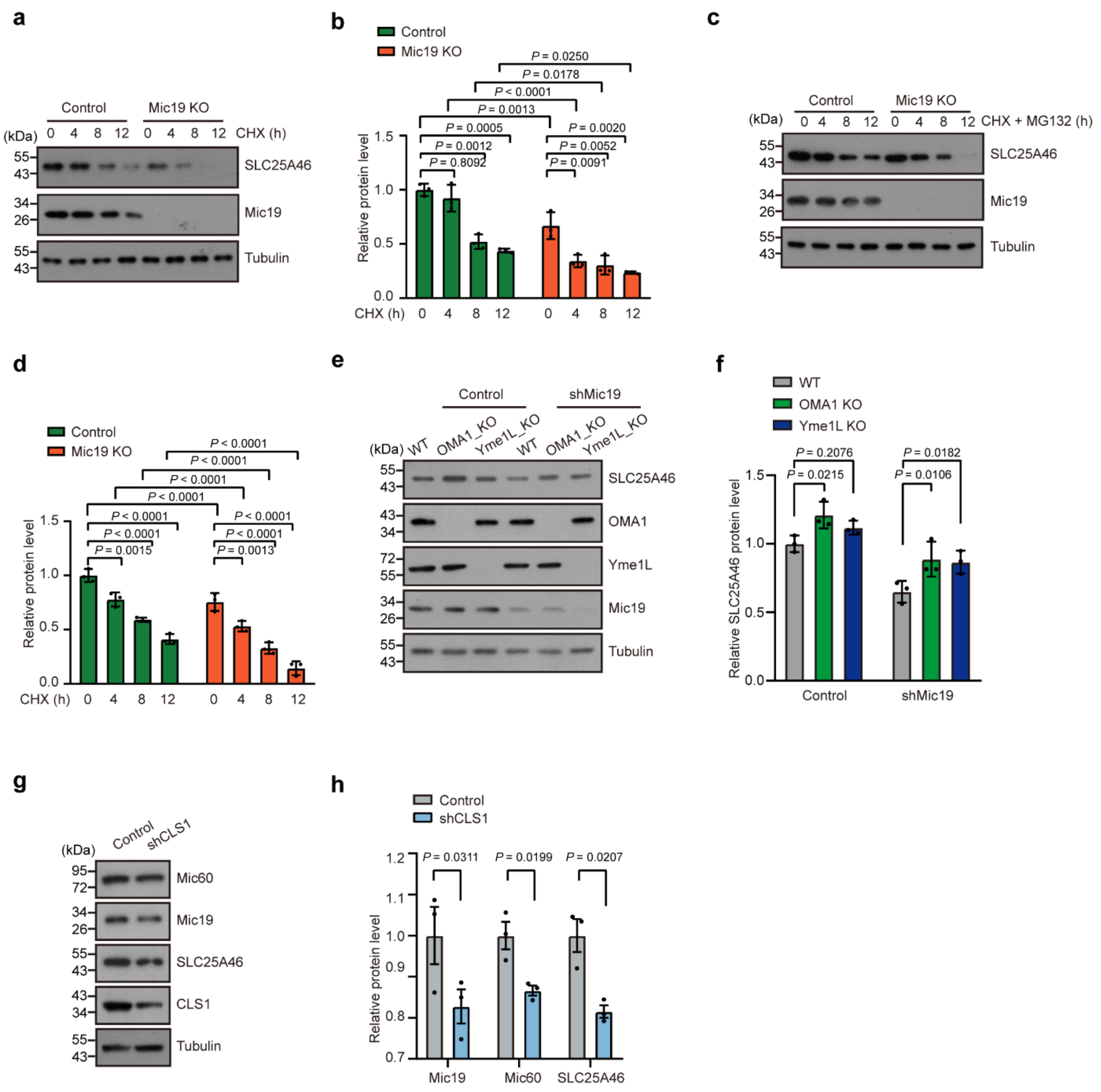

Figure S2. Mic19 depletion promotes degradation of SLC25A46 protein.

a-b Control and Mic19 KO Hela cells were treated with CHX (100 μg/ml) for 0, 4, 8, 12 h. Cell lysates were analyzed by Western blotting with antibodies against Mic19, SLC25A46, or Tubulin (**a**). Relative protein levels were further evaluated by densitometry analysis using ImageJ software (**b**). n = 3 independent experiments. Data are presented as mean ± SD, statistical significance was assessed by two-way ANOVA. **c-d** Control and Mic19 KO Hela cells were incubated with CHX (100 μg/ml) plus MG132 (10 μM) for 0, 4, 8, 12 h. Cell lysates were analyzed by Western blotting with antibodies against Mic19, SLC25A46, or Tubulin (**c**). Relative protein levels were further evaluated by densitometry analysis using ImageJ software (**d**). n = 3 independent experiments. Data are presented as mean ± SD, statistical significance was assessed by two-way ANOVA. **e-f** WT, OMA1_KO or Yme1L_KO HCT116 cells were

infected with control or shMic19 viral particles and then cultured for 5 days. Cell lysates were then subjected to Western blotting using the indicated antibodies (**e**). Relative protein levels were further evaluated by densitometry analysis using ImageJ software (**f**). n = 3 independent experiments. Data are presented as mean ± SD, statistical significance was assessed by two-way ANOVA. **g-h** Cell lysates of control or shCLS1 HeLa cells were analyzed by Western blotting with antibodies against Mic60, Mic19, SLC25A46, CLS1 or Tubulin (**g**). Representative immunoblots were from n = 3 independent experiments. Relative protein levels were further evaluated by densitometry analysis using ImageJ software (**h**). Results were representative of 3 independent experiments. Data are presented as mean ± SD, statistical significance was assessed by two-way ANOVA. *P* values are indicated in the figure. Source data are provided as a Source Data file.

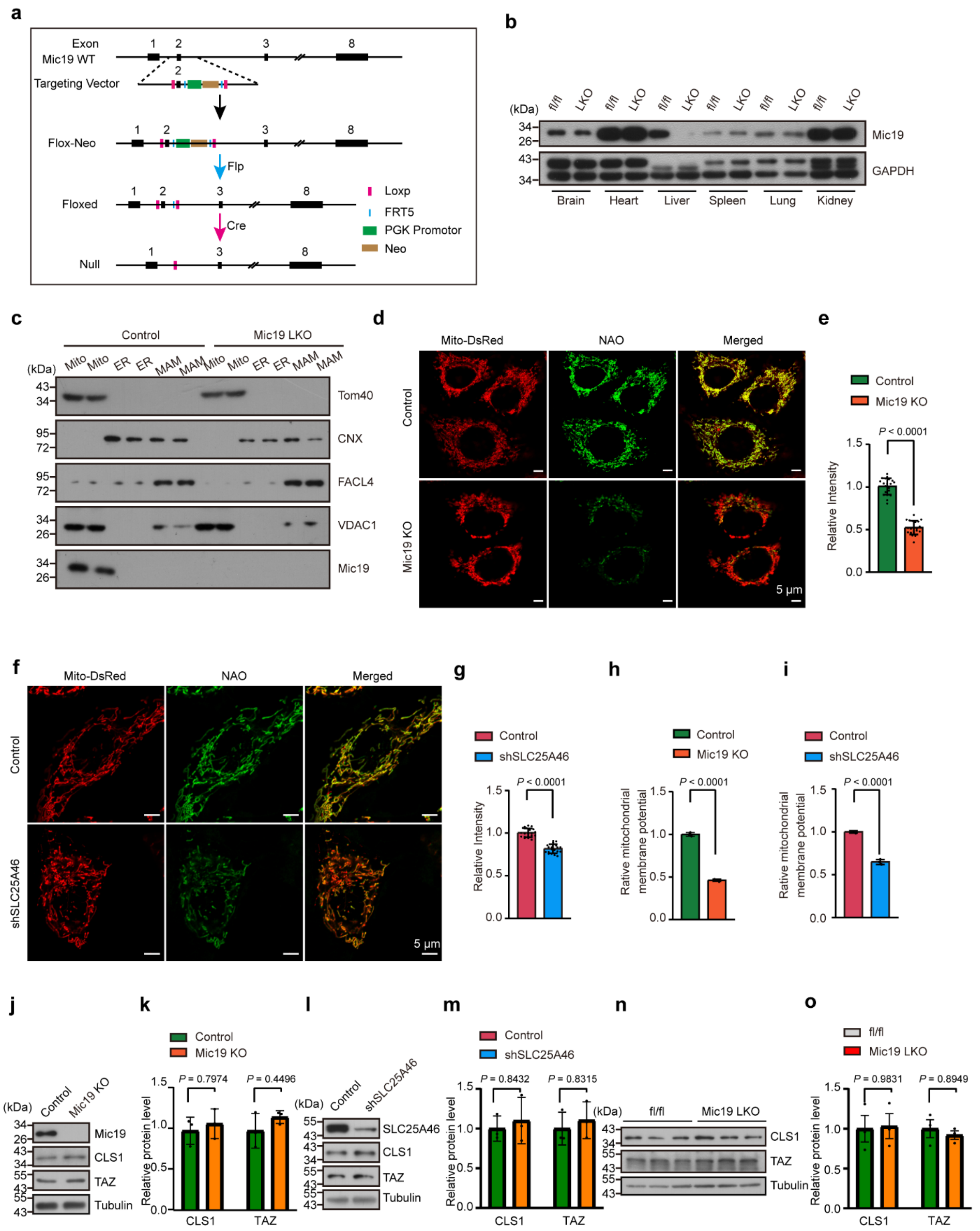

Figure S3. Mic19 or SCL25A46 depletion reduces CL level and mitochondrial membrane potential.

a Schematic of Cre mediated Mic19 ablation. Briefly, LoxP sequences were introduced on the side of Exon 2, resulting in the deletion of Exons 2 of the Mic19 gene in the presence of Cre. **b** Lysates of liver and other tissues (brain, heart, spleen, lung, kidney) from 3-month-old

Mic19$^{flox/flox}$ (control) or Mic19 LKO mice were analyzed by Western blotting with anti-Mic19 or anti-GAPDH antibodies. **c** Western blotting analysis of subcellular fractions prepared from control (Mic19$^{flox/flox}$) and Mic19 LKO mouse livers using the indicated antibodies. Mito: mitochondrial fraction; ER; MAM: mitochondria-associated membrane. **d-g** Control, Mic19 KO, or shSLC25A46 HeLa cells stably expressing Mito-DsRed were stained with NAO (10-N-Nonyl acridine orange), then imaged by a confocal microscope with Airyscan. (**d,f**). The relative fluorescence intensity was further assessed by ImageJ software (n = 20 cells) (**e, g**). 3 independent experiments were conducted. Data with error bars are presented as mean ± SD. Statistical significance was assessed by two-tailed Student's t-test. **h-i** Control, Mic19 KO (H) or shSLC25A46 (**i**) cells were stained with TMRM (200 nM), and then analyzed by Flow Cytometry for mitochondrial membrane potential. n= 3 independent experiments. Data with error bars are presented as mean ± SD. Statistical significance was assessed by two-tailed Student's t-tes. **j-o** Control and Mic19 KO (**j**), or control and shSLC25A46 (**l**) HeLa cell lysates or liver extracts isolated from 3-month-old Mic19$^{flox/flox}$ (control) or Mic19 LKO mice were analyzed by Western blotting with the indicated antibodies. Results shown are representative of 3 independent experiments. Relative protein levels (the indicated protein level/Tubulin level) were further assessed by densitometry analysis using ImageJ software (**k**, **m, o**). Data are shown as mean ± SD, statistical significance was assessed by two-way ANOVA. *P* values are indicated in the figure. Source data are provided as a Source Data file.

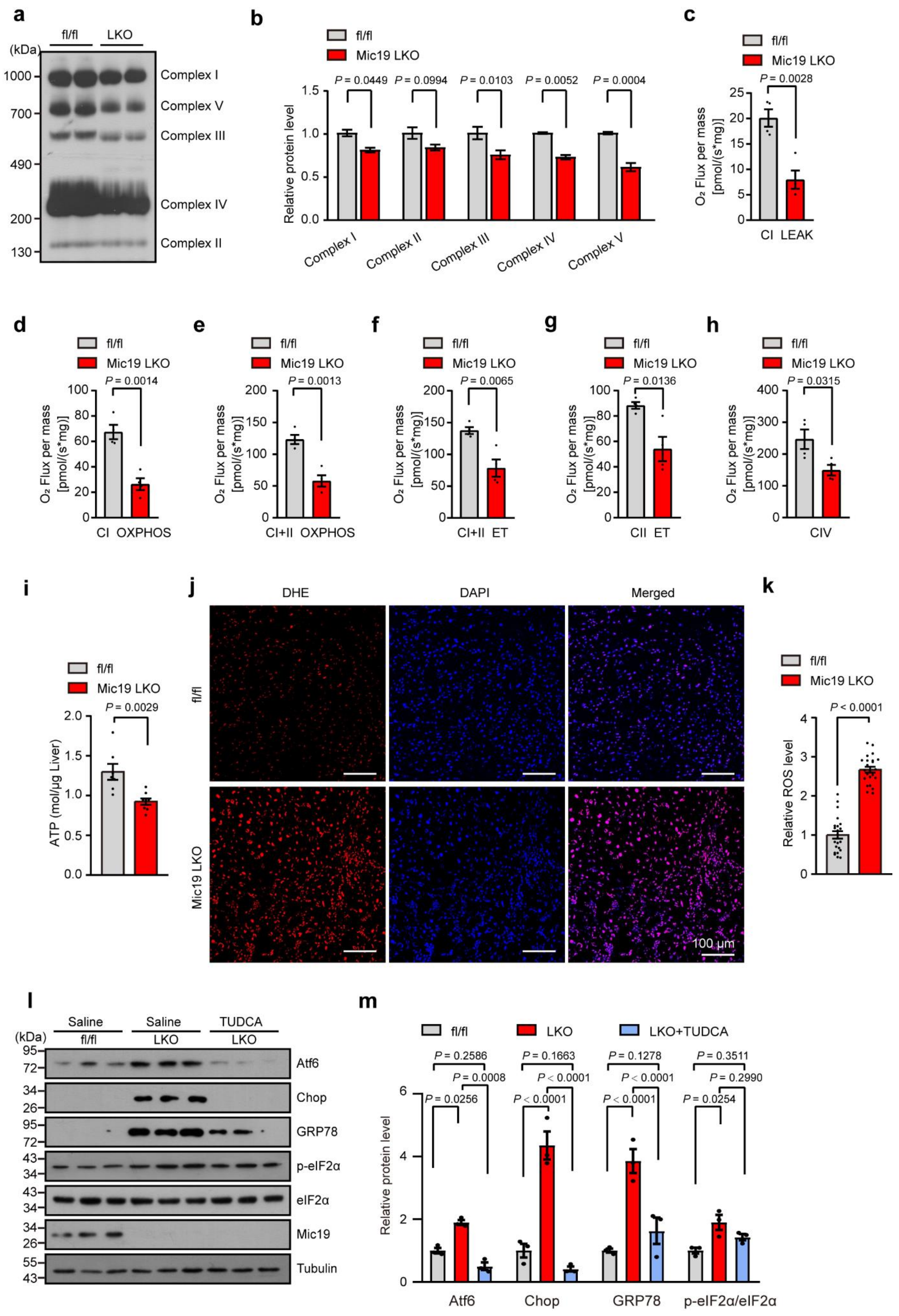

Figure S4. Mic19 LKO disrupts mitochondrial oxidative phosphorylation function in

mouse liver.

a-b Liver mitochondria isolated from 3-month-old Mic19$^{flox/flox}$ (control) or Mic19 LKO mice were used for blue native PAGE (BN-PAGE) analysis. Mitochondrial respiratory chain complexes were analyzed by Western blotting using antibodies against Complex I (NDUFB6), Complex II (SDHA), Complex III (UQCRC2), Complex IV (COX4) and Complex V (ATP5A1) (**a**). Relative complexes levels were further assessed by densitometry analysis using ImageJ software (**b**). Data are shown as mean ± SEM, statistical significance was assessed by two-way ANOVA. **c-h** Quantification of the respiratory states of mitochondria from 3-month-old Mic19$^{flox/flox}$ and Mic19 LKO mice (n = 4). CI LEAK (**c**), CI-driven leak respiration; CI OXPHOS (**d**), CI-driven phosphorylating respiration; CI+II OXPHOS (**e**), phosphorylating respiration driven by combined activation of CI and II; CI+II ET (**f**), electron transfer capacity driven by combined CI and II; CII ET (**g**), ET driven by CII; CIV (**h**), CIV-driven respiration; C, cytochrome c. Data are presented as mean ± SEM. Statistical significance was assessed by two-tailed Student's t-test**. i** ATP levels in liver of Mic19$^{flox/flox}$ (control, n = 7) or Mic19 LKO (n = 8) mice at 3 months were measured. Data are mean ± SEM. Statistical significance was assessed by two-tailed Student's t-test. **j-k** Dihydroethidium (DHE) staining of liver tissues from 3-month-old Mic19$^{flox/flox}$ or Mic19 LKO mice was conducted. Representative images of 3 independent experiments were shown (**j**). Relative ROS level was analyzed by ImageJ software (**k**). n = 23 fields. Data are shown as mean ± SEM, statistical significance was assessed by two-tailed Student's t-test. **l-m** The 3-month-old Mic19 LKO mice were intraperitoneally injected with TUDCA (100 mg/kg/day for consecutive 14 days). Then liver extracts from control, Mic19 LKO and TUDCA-injected mice were analyzed by Western blotting analysis using the indicated antibodies (**l**). Relative protein levels (the indicated protein level/Tubulin level) were further evaluated by densitometry analysis using ImageJ software (**m**). n= 3 mice Data are presented as mean ± SEM, statistical significance was assessed by two-way ANOVA. *P* values are indicated in the figure. Source data are provided as a Source Data file.

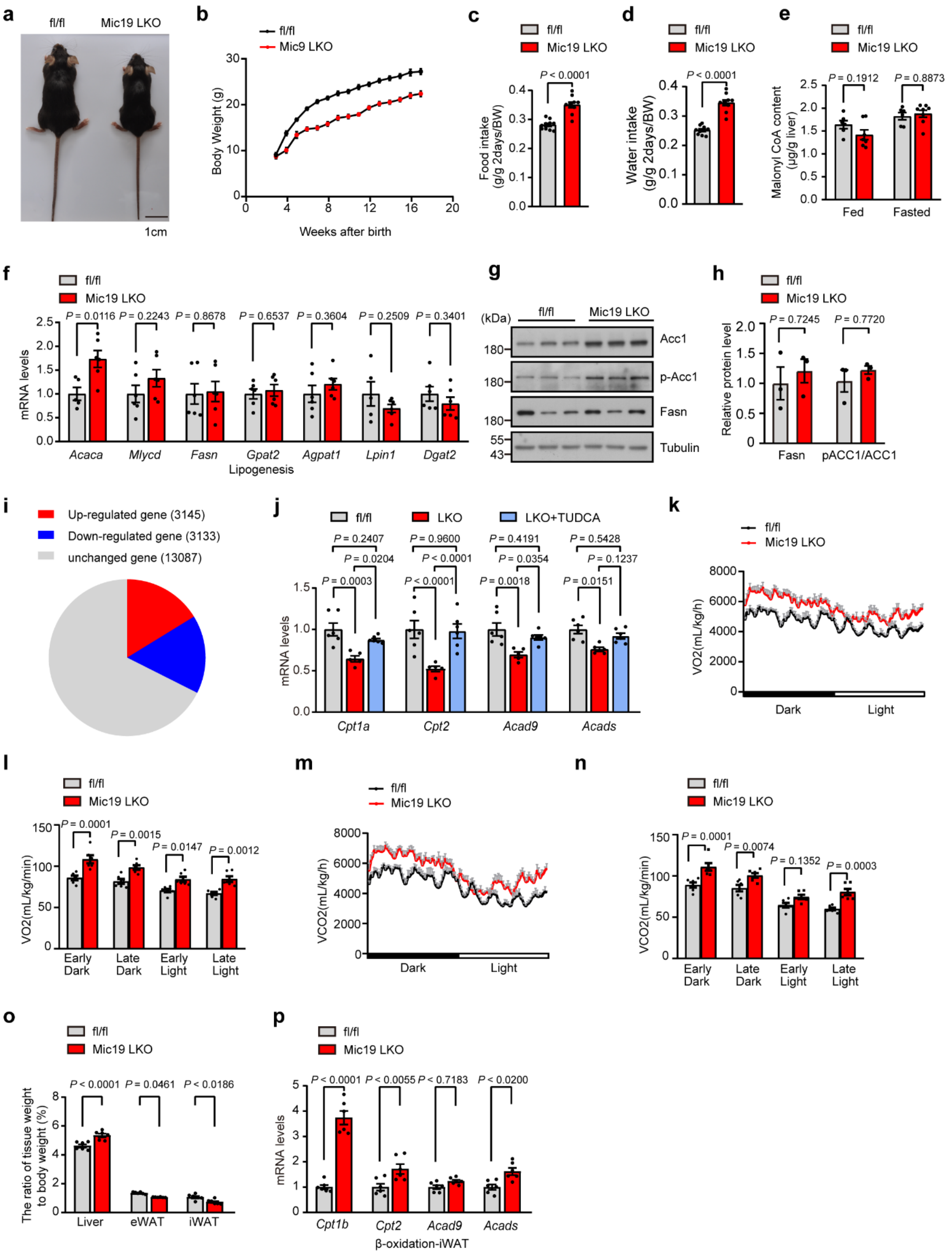

Figure S5. Mic19 LKO causes abnormal energy metabolism in mice.

a Representative images of 3-month-old Mic19$^{flox/flox}$ (control)and Mic19 LKO mice. **b** Body weight of Mic19$^{flox/flox}$ and Mic19 LKO mice were collected once a week (n = 8 mice). **c-d** Food (**c**) and water intake (**d**) over two days were assessed in Mic19$^{flox/flox}$ (control) or Mic19

LKO mice aged 3 months (n = 10 mice). **e** Malonyl-CoA level in livers isolated from fed and 24-hour-fasted Mic19$^{flox/flox}$ (n = 5 mice) or Mic19 LKO mice aged at 3 months (n = 8 mice) were detected. **f, j p** Relative mRNA level of lipogenesis related genes (**f**) and fatty acid β-oxidation related genes(**p**) in livers isolated from Mic19$^{flox/flox}$ (n = 5 mice) or Mic19 LKO mice (n = 6 mice) and the relative mRNA level of fatty acid β-oxidation related genes of Mic19$^{flox/flox}$, Mic19 LKO and TUDCA-injected mice to GAPDH were measured by quantitative RT-PCR analysis. **g-h** The relative protein levels of Acc1, p-Acc1, Fasn in 3-month-old Mic19$^{flox/flox}$ (control) or Mic19 LKO mouse livers were detected(**g**) and were evaluated by densitometry analysis using ImageJ software (**h**). **i** The schematic pie chart indicates statistically altered (fold change > 1.2 and *P*-Value < 0.05) genes by Mic19 ablation. **k-n** Oxygen consumption (VO2) or Carbon dioxide production (VCO2) of 9-week-old Mic19$^{flox/flox}$ (control) and Mic19 LKO mice (n = 6 mice) were detected. Real-time statistics data (**k, m**) and the analyzed data in different time (**l, n**) were shown. **o** Liver, eWAT or iWAT weight of Mic19$^{flox/flox}$ and Mic19 LKO mice in 3 months old (n = 6 mice). Data in **Figure S5** are means ± SEM. Statistical significance in Fig. **c-d** was assessed by two-tailed Student's t-test. Statistical significance in Fig. **e, f, h, l, n, o** and **p** performed by two-way ANOVA. *P* values are indicated in the figure. Source data are provided as a Source Data file.

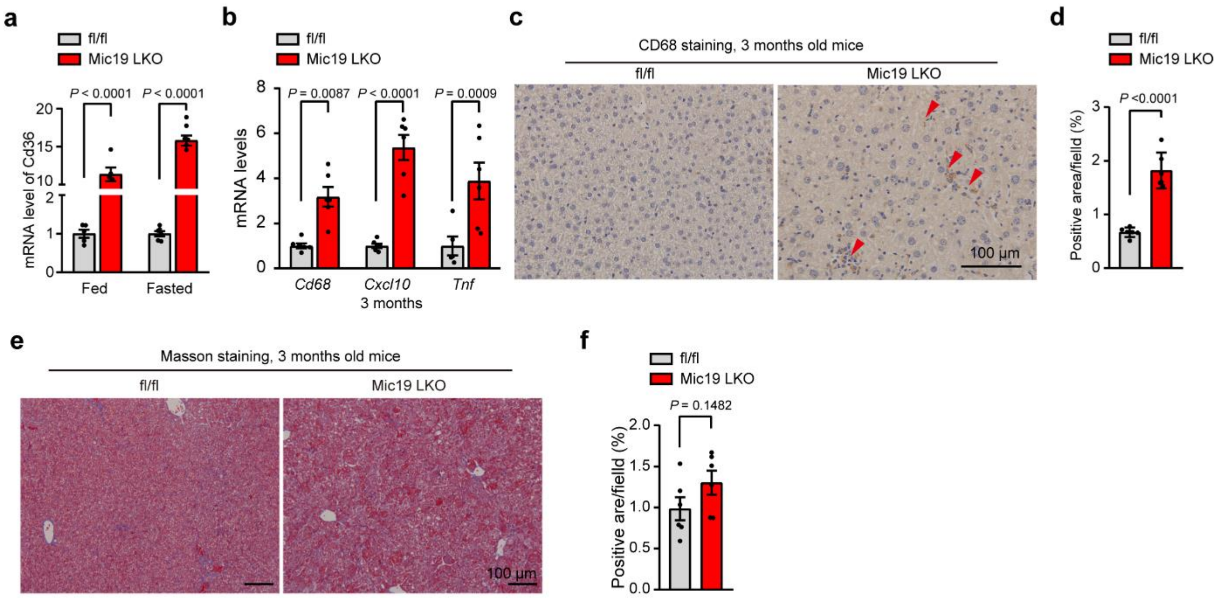

Figure S6. Mic19 LKO develops a NASH-like phenotype at early stage in mice.

a Relative mRNA level of *Cd36* in fed and 24-hour-fasted Mic19$^{flox/flox}$ (control, n = 4 mice) or Mic19 LKO mice aged 3 months (n = 6 mice) to GAPDH were measured by quantitative RT-PCR. Data are presented as mean ± SEM; statistical analysis was assessed using two-way ANOVA. **b** Relative liver mRNA levels of inflammatory cytokines (*Cd68, Cxcl10, Tnf*) from 3-month-old Mic19$^{flox/flox}$ (n = 5 mice) or Mic19 LKO mice (n= 6 mice) to GAPDH were determined by quantitative RT-PCR. Data are presented as mean ± SEM; statistical analysis was assessed using two-way ANOVA. **c-f** Liver tissues from Mic19$^{flox/flox}$ (control) or Mic19 LKO mice aged 3 months were fixed with formaldehyde, and the liver sections were used for CD68 (**c**) and Masson's trichrome staining (**e**). Representative images of three independent experiments were shown. Liver damaged areas in CD68 staining images (**d**) and Masson's trichrome staining images (**f**) were analyzed and quantified by ImageJ software. Data are shown as mean ± SEM, statistical significance was assessed by two-tailed Student's t-test. *P* values are indicated in the figure. Source data are provided as a Source Data file.

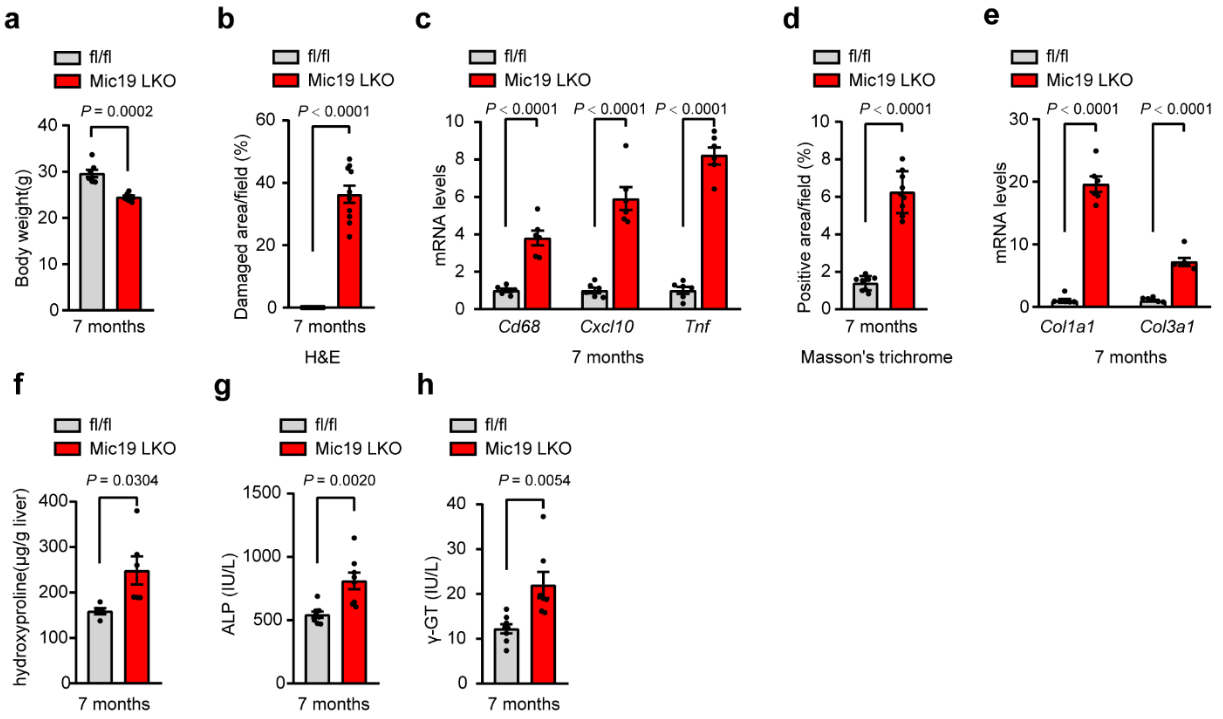

Figure S7. Mic19 LKO develops hepatic liver fibrosis at later stage in mice.

a Body weight of littermate Mic19$^{flox/flox}$ (control, n = 6 mice) or Mic19 LKO mice at 7 months old (n= 7 mice). Data are mean ± SEM; statistical significance was assessed by two-tailed Student's t-test. **b** Liver damaged areas in H&E staining images were analyzed and quantified by ImageJ software. Data are shown as mean ± SEM, statistical significance was assessed by two-tailed Student's t-test. **c** Relative liver mRNA levels of inflammatory cytokines (*Cd68, Cxcl10, Tnf*) were determined by quantitative RT-PCR analysis from 7-month-old Mic19$^{flox/flox}$ (control) or Mic19 LKO mice (n = 6 mice). The mRNA levels of target genes were normalized to that of GAPDH. Data are presented as mean ± SEM; statistical analysis was assessed using two-way ANOVA. **d** Liver damaged areas in Masson's trichrome staining were analyzed and quantified by ImageJ software. Data are shown as mean ± SEM, statistical significance was assessed by two-tailed Student's t-test. **e** The expression of collagen related genes (*Col1a1, Col3a1*) in liver tissues from 7-month-old Mic19$^{flox/flox}$ (control) and Mic19 LKO mice (n = 6 mice) were detected by quantitative RT-PCR analysis. Data are presented as mean ± SEM, statistical significance was evaluated by two-way ANOVA. **f** Hydroxyproline level in livers isolated from 7-month-old Mic19$^{flox/flox}$ (control, n = 5) or Mic19 LKO mice were detected (n = 6). Data are mean ± SEM, statistical significance was evaluated by two-tailed Student's t-test. **g-h** Alkaline phosphatase (ALP) (**g**) and γ-glutamyl transpeptidase (γ-GT) (**h**) activities of Mic19$^{flox/flox}$ (n = 7 mice) or Mic19 LKO mice (n = 8 mice) at 7 months old were analyzed. The

data are shown as mean ± SEM, statistical significance was evaluated by Student's t-test, two-tailed. *P* values are indicated in the figure. Source data are provided as a Source Data file.

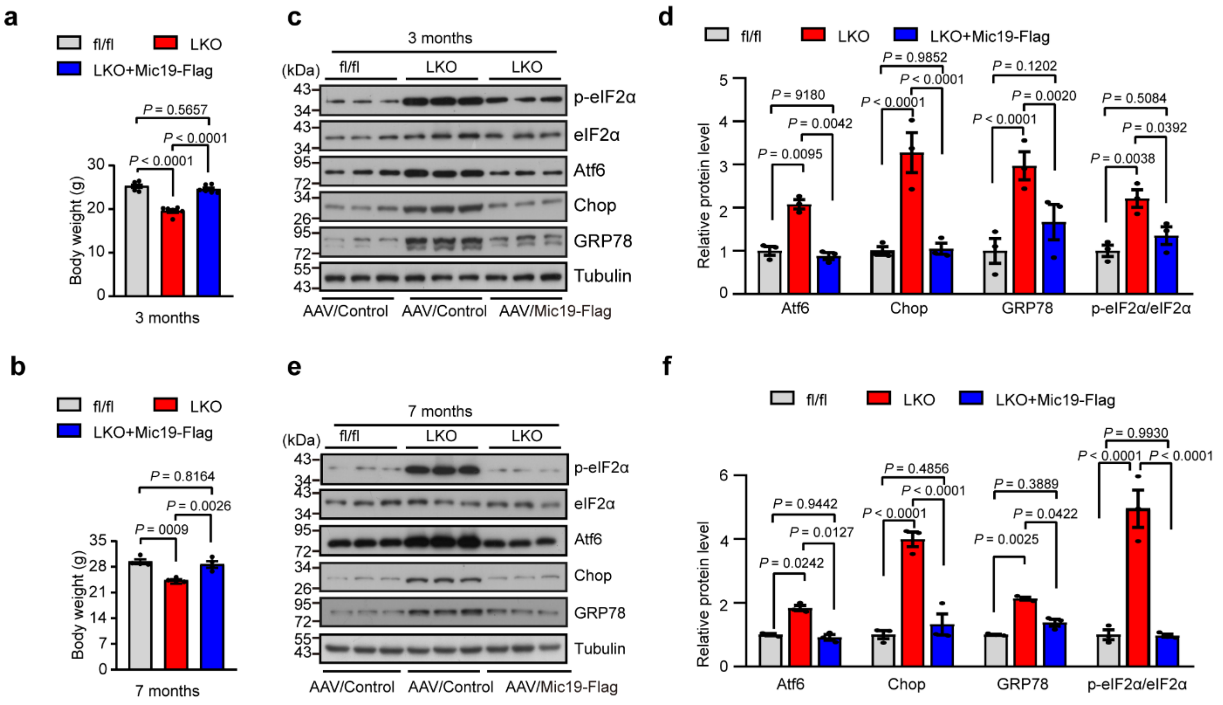

Figure S8. Mic19 re-expression attenuates ER stress in Mic19 LKO mice.

a-b Body weight was measured from 3-month-old (**a**) and 7-month-old (**b**) Mic19$^{flox/flox}$ (control), Mic19 LKO, or Mic19 re-expression Mic19 LKO mice (n = 6 mice). Data are shown as mean ± SEM, statistical significance was assessed by one-way ANOVA. **c-f** Western blotting analysis of liver lysates isolated from 3-month-old (**c**) or 7-month-old (**e**) Mic19$^{flox/flox}$ (control), Mic19 LKO and Mic19 re-expression Mic19 LKO mice using antibodies against p-eIF2α, eIF2α, Atf6, Chop, GRP78 (ER stress markers), or Tubulin (**c** and **e**). Relative protein levels (the indicated protein level/Tubulin level) were evaluated by densitometry analysis using ImageJ software (**d** and **f**). Data with error bars are presented as mean ± SEM. Statistical significance was assessed by two-way ANOVA. *P* values are indicated in the figure. Source data are provided as a Source Data file.

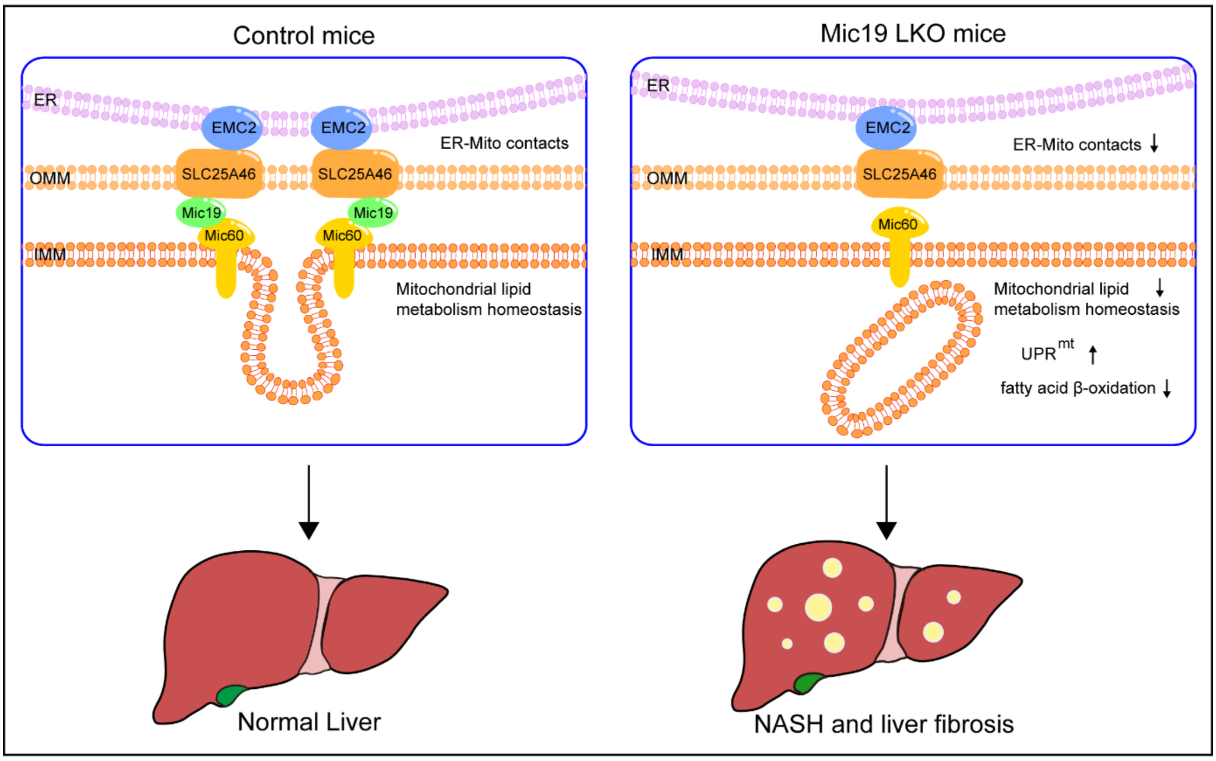

Figure S9. The mode of Mic19 in regulating ER-mitochondrial PA transport and liver disease.

The Mic19-SLC25A46-EMC2 axis regulates mitochondria-ER contacts and mitochondrial lipid metabolism, then impairing mitochondrial membrane organization and inducing UPR^{mt}, leading to impaired fatty acid metabolism, and resulting in NASH and liver fibrosis in mice.
