# Supplementary material for: Mic19 depletion impairs endoplasmic reticulum-mitochondrial contacts and mitochondrial lipid metabolism and triggers liver disease
Source: Nat Commun. 2024 Jan 2;15:168. doi: 10.1038/s41467-023-44057-6 (PMC10762189; doi:10.1038/s41467-023-44057-6)
Supplement: Supplementary file 3 — Reporting Summary [file 41467_2023_44057_MOESM3_ESM.pdf]

nature portfolio

Corresponding author(s): Zhiyin Song, He He

Last updated by author(s): Nov 20, 2023

Reporting Summary

Nature Portfolio wishes to improve the reproducibility of the work that we publish. This form provides structure for consistency and transparency in reporting. For further information on Nature Portfolio policies, see our Editorial Policies and the Editorial Policy Checklist.

Statistics

For all statistical analyses, confirm that the following items are present in the figure legend, table legend, main text, or Methods section.

n/a	Confirmed	
☐	☒	The exact sample size (*n*) for each experimental group/condition, given as a discrete number and unit of measurement
☐	☒	A statement on whether measurements were taken from distinct samples or whether the same sample was measured repeatedly
☐	☒	The statistical test(s) used AND whether they are one- or two-sided<br>*Only common tests should be described solely by name; describe more complex techniques in the Methods section.*
☒	☐	A description of all covariates tested
☒	☐	A description of any assumptions or corrections, such as tests of normality and adjustment for multiple comparisons
☐	☒	A full description of the statistical parameters including central tendency (e.g. means) or other basic estimates (e.g. regression coefficient) AND variation (e.g. standard deviation) or associated estimates of uncertainty (e.g. confidence intervals)
☐	☒	For null hypothesis testing, the test statistic (e.g. *F*, *t*, *r*) with confidence intervals, effect sizes, degrees of freedom and *P* value noted<br>*Give P values as exact values whenever suitable.*
☒	☐	For Bayesian analysis, information on the choice of priors and Markov chain Monte Carlo settings
☒	☐	For hierarchical and complex designs, identification of the appropriate level for tests and full reporting of outcomes
☒	☐	Estimates of effect sizes (e.g. Cohen's *d*, Pearson's *r*), indicating how they were calculated

Our web collection on statistics for biologists contains articles on many of the points above.

Software and code

Policy information about availability of computer code

Data collection	All SIM images were collected using HIS-SIM (High Sensitivity Structured Illumination Microscope) controlled by HIS-SIM IMAGER software (Guangzhou Computational Super-resolution Biotech, V1.1.24a). Confocal imaging was collected using ZEISS LSM880 laser scanning confocal microscope controlled by Zeiss Zen software equipped with Airyscan module and Leica SP8 controlled by LAS X software (version 3.3, Leica and version LAS4.13). Lipidomic data was collected on Liquid Chromatography system using a Shimadzu Nexera 20AD-HPLC coupled with Sciex QTRAP 6500 PLUS via an electrospray ionization source (ESI) with positive and negative ionization mode. mRNA-seq libraries were sequenced using HiSeq2000 for 100-bp paired-end sequencing, and the data were mapped to hg19 genome reference by Tophat2. The images were collected a JEM-1400Plus (JEOL) transmission electron microscope at an acceleration voltage of 100 kV. Target mRNA expression were used the iCycler real-time PCR Detection System (Bio-Rad CFX Connect) with CFXMaestroSetup1.0. Flow analysis was performed using a Beckman flow cytometer with CytExpert Software 2.4 and FlowJo7.6.1.
Data analysis	Statistical analyses were performed using GraphPad Prism 8.0.Genes with fold change > 1.2 and p-Value < 0.05 were considered as significantly changed. Gene ontology (GO) analysis was performed using KOBAS 3.0 website. The distance between mitochondria and ER, lipid droplet size, etc. were analyzed by ImageJ 1.48v software. Lipidomic data was analyzed by Z-Score, and processed for heatmap production using MeV v4.8.1 software. The fold change of target mRNA expression was calculated using the 2-ΔΔCT method. The fluorescence intensity of Mitochondrial ROS was analyzed by ImageJ 1.48v software.<br>The co-localization of ER and mitochondria were analyzed by ImageJ 1.48v software. Western blotting analysis of the relative protein were evaluated by densitometry analysis using ImageJ 1.48v software.

For manuscripts utilizing custom algorithms or software that are central to the research but not yet described in published literature, software must be made available to editors and reviewers. We strongly encourage code deposition in a community repository (e.g. GitHub). See the Nature Portfolio guidelines for submitting code & software for further information.

Data

Policy information about availability of data

All manuscripts must include a data availability statement. This statement should provide the following information, where applicable:

- Accession codes, unique identifiers, or web links for publicly available datasets
- A description of any restrictions on data availability
- For clinical datasets or third party data, please ensure that the statement adheres to our policy

All the data and relevant materials, including reagents and primers, that support the findings of this study are available from the corresponding author upon reasonable request.

Human research participants

Policy information about studies involving human research participants and Sex and Gender in Research.

Reporting on sex and gender	Our research content dose not cover this field work.
Population characteristics	Our research content dose not cover this field work.
Recruitment	Our research content dose not cover this field work.
Ethics oversight	Our research content dose not cover this field work.

Note that full information on the approval of the study protocol must also be provided in the manuscript.

Field-specific reporting

Please select the one below that is the best fit for your research. If you are not sure, read the appropriate sections before making your selection.

- [x] Life sciences
- [ ] Behavioural & social sciences
- [ ] Ecological, evolutionary & environmental sciences

For a reference copy of the document with all sections, see nature.com/documents/nr-reporting-summary-flat.pdf

Life sciences study design

All studies must disclose on these points even when the disclosure is negative.

Sample size	No statistic method was used to predetermine sample size. For most in vitro experiments, at least 3 independent biological replicates were conducted. The sample size of all mouse experiments should be at least 3 for each group.
Data exclusions	No data were excluded from the analyses.
Replication	All of experiments were performed at least three times. Experimental results are reliably reproduced.
Randomization	The experiments with cell samples and mice were allocated into experimental groups with different treatments.
Blinding	For co-localization between ER and mitochondria, a blinded quantification was applied. At least 3 independent experiments were performed for the analysis. The distance between the ER and mitochondria, mitochdrial length and number of tight ER-mito contact in EM images were analyzed by ImageJ software and the analysis was performed in a double-blind fashion. At least 3 independent experiments were conducted. Statistical analysis was performed using Prism 8 (GraphPad Software).

Reporting for specific materials, systems and methods

We require information from authors about some types of materials, experimental systems and methods used in many studies. Here, indicate whether each material, system or method listed is relevant to your study. If you are not sure if a list item applies to your research, read the appropriate section before selecting a response.

Materials & experimental systems

n/a	Involved in the study
☐	☒ Antibodies
☐	☒ Eukaryotic cell lines
☒	☐ Palaeontology and archaeology
☐	☒ Animals and other organisms
☒	☐ Clinical data
☒	☐ Dual use research of concern

Methods

n/a	Involved in the study
☒	☐ ChIP-seq
☐	☒ Flow cytometry
☒	☐ MRI-based neuroimaging

Antibodies

Antibodies used

Mic19 (Proteintech, 25625-1-AP), Mic60 (Proteintech, 10179-1-AP), Mic10 (Origene, TA505025), EMC2 (Proteintech, 25443-1-AP), SLC25A46 (Proteintech, 12277-1-AP), Flag (Sigma-Aldrich, F1804), GAPDH (Santa Cruz, sc-166545), Mic13 (Sigma-Aldrich, SAB1102836), Tom40 (Proteintech, 18409-1-AP), β-Tubulin (GNI, GNI4110-BT), LONP1 (Proteintech, 15440-1-AP), ClpP (Proteintech, 15698-1-AP), HSP60 (Abclonal, A0969), SOD2 (Proteintech, 24127-1-AP), Atf6 (Proteintech, 24169-1-AP), Chop (Cell Signaling Technology, #5554), GRP78 (Proteintech, 11587-1-AP), eIF2α (Cell Signaling Technology, #5324), phospho-eIF2α(Ser-51) (Cell Signaling Technology, #3398), ACC1 (Proteintech, 21923-1-AP), phospho-ACC1 (-S79) (Abclonal, AP0298); FASN (Proteintech, 10624-2-AP), CLS1 (Proteintech, 51055-1-AP), TAZ (Proteintech, 23306-1-AP), NDUFB6 (Proteintech, 16037-1-AP), SDHA (Proteintech, 14865-1-AP), UQCRC2 (Proteintech, 14742-1-AP), COX2 (Proteintech, 55070-1-AP), ATP5A1 (Proteintech, 14676-1-AP), FACL4 (Abcam, Cat# ab155282), CNX (Proteintech, 81938-1-RR), Tom20 (Proteintech, 66777-1-Ig), Tim23 (Proteintech, 11123-1-AP), Cox4 (Proteintech, 66110-1-Ig), VDAC1 (Proteintech, 55259-1-AP), Oma1 (Proteintech, 17116-1-AP), Yme1L (Proteintech, 11510-1-AP).

Validation

Antibody (Supplier, Cat. No) Dilution factor

Mic19 (Proteintech, 25625-1-AP)1 : 3000/Manufacturer COA detected Mic19 in HeLa cells and human liver (Western and Immunohistochemistry), Mic60 (Proteintech, 10179-1-AP)1 : 3000/Manufacturer COA detected Mic60 in HepG2 cells and HeLa cells (Western and Immunofluorescencence), Mic10 (Origene, TA505025)1 : 1000/Manufacturer COA detected Mic10 in HeLa cells and Human breast tissue (Western and Immunohistochemistry), SLC25A46 (Proteintech, 12277-1-AP)1 : 3000/Manufacturer COA detected SLC25A46 in Jurkat cells and mouse brain (Western), Flag (Sigma-Aldrich, F1804)1 : 3000/Manufacturer COA detected Flag tagged myr-PKCz in MDCK canine kidney epithelial cell (Immunofluorescencence), GAPDH (Santa Cruz, sc-166545)1 : 5000/Manufacturer COA detected GAPDH in Hep G2 (Western), EMC2 (Proteintech, 25443-1-AP)1 : 3000/Manufacturer COA detected EMC2 in mouse heart tissue (Western),Mic13 (Sigma-Aldrich, SAB1102836)1 : 2000/Manufacturer COA detected Mic13 in HEK-293T (Western), Tom40 (Proteintech, 18409-1-AP)1 : 5000/Manufacturer COA detected Tom40 in HeLa and human liver (Western, Immunofluorescence), β-Tubulin (Proteintech, 10094-1-AP) 1 : 6000/Manufacturer COA detected β-Tubulin in Hep G2 and mouse brain (Western and Immunohistochemistry), LONP1 (Proteintech, 15440-1-AP)1 : 3000/Manufacturer COA detected LONP1 in HeLa and human lung cancer (Western and Immunohistochemistry), ClpP (Proteintech, 15698-1-AP)1 : 4000/Manufacturer COA detected ClpP in HeLa and human liver cancer (Western, Immunofluorescence and Immunohistochemistry), HSP60 (Abclonal, A0969)1 : 5000/Manufacturer COA detected HSP60 in HeLa and rat kidney (Western, Immunofluorescencence and Immunohistochemistry), SOD2 (Proteintech, 24127-1-AP)1 : 5000/Manufacturer COA detected SOD2 in HeLa and human liver cancer (Western and Immunohistochemistry), Atf6 (Proteintech, 24169-1-AP)1 : 5000/Manufacturer COA detected Atf6 in HeLa and human cervucal cancer tissue (Western, Immunofluorescence and Immunohistochemistry), Chop (Cell Signaling Technology, #5554)1 : 3000/Manufacturer COA detected Chop in C2C12 cells (Western), GRP78 (Proteintech, 11587-1-AP)1 : 3000/Manufacturer COA detected GRP78 in HeLa cells, HepG2 cells and human breast cancer tissue(Western and Immunohistochemistry), eIF2α (Cell Signaling Technology, #5324)1 : 3000/Manufacturer COA detected eIF2α in HeLa cells and human lung cancer tissue(Western and Immunohistochemistry), phospho-eIF2α(Ser-51) (Cell Signaling Technology, #3398)1 : 3000/Manufacturer COA detected phosphor-eIF2α in C2C12 cells, and human lung cancer tissue(Western and Immunohistochemistry), ACC1 (Proteintech, 21923-1-AP)1 : 3000/Manufacturer COA detected ACC1 in HeLa cells and mouse brain tissue (Western and Immunohistochemistry), phospho-ACC1 (-S79) (Abclonal, AP0298)1 : 3000/Manufacturer COA detected phospho-ACC1 (-S79) in RAW264.7 cells (Western); FASN (Proteintech, 10624-2-AP)1:3000/Manufacturer COA detected FASN in HeLa cells and mouse liver tissue, (Western and Immunohistochemistry), CLS1 (Proteintech, 51055-1-AP)1:3000/Manufacturer COA detected CLS1 in mouse and human heart tissue (Western and Immunohistochemistry), TAZ (Proteintech, 23306-1-AP)1:3000/Manufacturer COA detected TAZ in HeLa cells and rat kidney tissue (Western and Immunohistochemistry), NDUFB6 (Proteintech, 16037-1-AP)1 : 5000/Manufacturer COA detected NDUFB6 in MCF-7 cells and human liver cancer tissue (Western and Immunohistochemistry), SDHA (Proteintech, 14865-1-AP)1 : 5000/Manufacturer COA detected SDHA in HeLa cells and rat brain tissue (Western and Immunohistochemistry), UQCRC2 (Proteintech, 14742-1-AP)1 : 5000/Manufacturer COA detected UQCRC2 in mouse colon tissue and mouse heart tissue (Western and Immunohistochemistry), COX2 (Proteintech, 55070-1-AP)1 : 2500/Manufacturer COA detected COX2 in HeLa cells and mouse liver (Western and Immunohistochemistry), ATP5A1 (Proteintech, 14676-1-AP)1 : 5000/Manufacturer COA detected ATP5A1 in HeLa cells, (Western, Immunofluorescence and Immunohistochemistry), FACL4 (Abcam, Cat# ab155282)1 : 3000/Manufacturer COA detected FACL4 human hepatocellular carcinoma tissue (Western), CNX (Proteintech, 81938-1-RR)1 : 5000/Manufacturer COA detected CNX in HeLa cells (Western), Tom20 (Proteintech, 66777-1-Ig)1 : 5000/Manufacturer COA detected Tom20 in HeLa cells and human liver cancer tissue (Western, Immunofluorescence and Immunohistochemistry), Tim23 (Proteintech, 11123-1-AP)1 : 3500/Manufacturer COA detected Tim23 in HepG2 cells (Western, Cox4 (Proteintech, 66110-1-Ig)1 : 2500/Manufacturer COA detected Cox4 in HeLa cells and human prostate cancer tissue (Western), VDAC1 (Proteintech, 55259-1-AP)1 : 3000/Manufacturer COA detected VDAC1 in HepG2 cells (Western), Oma1 (Proteintech, 17116-1-AP)1 : 2000/Manufacturer COA detected Oma1 in HeLa cells (Western), Yme1L (Proteintech, 11510-1-AP)1 : 2000/Manufacturer COA detected Yme1L in HeLa cells (Western).

Eukaryotic cell lines

Policy information about cell lines and Sex and Gender in Research

Cell line source(s)	Human cervical cancer HeLa cell line (ATCC), Monkey kidney COS-7 cell line (ATCC) and Human embryonic kidney 293T cells (ATCC) were grown in DMEM (Invitrogen) supplemented with 10% fetal bovine serum (Gibco) and 1% penicillin/streptomycin.
Authentication	All cell lines used in this study was authenticated by the provider.
Mycoplasma contamination	All of the cell lines used in this study are free of mycoplasma contamination.
Commonly misidentified lines (See ICLAC register)	No commonly misidentified cell lines were used.

Animals and other research organisms

Policy information about studies involving animals; ARRIVE guidelines recommended for reporting animal research, and Sex and Gender in Research

Laboratory animals	For MCD experiment, 8-week-old C57BL/6 mice were fed a standard of methionine choline-deficient diet (MCD) with 45% high fat diet (HFD) and supplemented with 0.1% L-methionine in drinking water for 5 weeks. Mic19 floxed mice were generated by conventional gene targeting of mouse embryonic stem cells (ESC) derived from C57BL/6 mice using the Mic19 gene targeting constructs designed to insert LoxP sites flanking exon 2 of Mic19 gene. For generation of Mic19 liver specific knockout (LKO) mice, Mic19 floxed mice were crossed with Alb-Cre transgenic mice. The male 8-week-old Mic19 LKO mice were injected with adeno-associated virus expressing control or Mic19-Flag via tail vein. All animals in this study were males.
Wild animals	Our research dose not cover wild animals.
Reporting on sex	The mouse phenotypes were observed in this manuscript apply to both sexes.
Field-collected samples	Our research does not involve field-collected samples.
Ethics oversight	All animal experiments were performed according to the guidelines of the China Animal Welfare Legislation and Use Committee of Wuhan University.

Note that full information on the approval of the study protocol must also be provided in the manuscript.

Flow Cytometry

Plots

Confirm that:

- [x] The axis labels state the marker and fluorochrome used (e.g. CD4-FITC).
- [x] The axis scales are clearly visible. Include numbers along axes only for bottom left plot of group (a 'group' is an analysis of identical markers).
- [ ] All plots are contour plots with outliers or pseudocolor plots.
- [x] A numerical value for number of cells or percentage (with statistics) is provided.

Methodology

Sample preparation	Digested HeLa cells were successively treated with 2% PFA, and NAO staining.
Instrument	Beckman
Software	CytExpert 2.4,FlowJo_V10
Cell population abundance	10,000 single cells were counted and the FITC average was recorded by flow cytometry.
Gating strategy	Firstly, the live cells were first circled in the scatter plot of FSC-A and SSC-A. Then single cells were circled by FSC-H and FSC-A scatter plot for further fluorescence intensity analysis.

- [ ] Tick this box to confirm that a figure exemplifying the gating strategy is provided in the Supplementary Information.
